# Self-immolation Among Medical Practitioners and Medical Students: More Evidence is Needed from Developing Countries

**Published:** 2014

**Authors:** Mohsen Rezaeian

**Affiliations:** 1 Department of Social Medicine, Occupational Environmental Research Center AND Rafsanjan Medical School, Rafsanjan University of Medical Sciences, Rafsanjan, Iran

Evidence suggests the high rates of “burnout,” “depression,” and “suicidal ideation” among medical doctors and medical students. However, due to privacy, possible job impact, stigma, etc. these two groups might not seek proper mental health treatment (1). As a result, medical practitioners and medical students have a higher suicide rate compared with the general population (2-7). It has been suggested that familiarity with and easy access to dangerous means of suicide such as drugs could partially explain this elevated risk (8, 9).

For example, the results of a study that carried out in England and Wales have highlighted that using drugs was common in doctors in comparison with the general population. During the period of study, that is, 1979-1995, 115 (54.8%) male doctors and 40 (64.5%) female doctors committed suicide applying drugs or poisoning. Interestingly, the same study also revealed that only 2 (2.3%) male doctors and no female doctors committed suicide applying self-burning (10). Furthermore, the results of another study have demonstrated that doctors in a general hospital medicine have lower rates compare with anesthetists and psychiatrists (11).

However, the problem with above studies is that almost all of them were carried out in the developed countries and very few studies originated from developing countries (12). As a result, evidence is lacking regarding suicide methods applied in medical practitioners and medical students in developing countries, especially from those countries such as India, Iran, Sri Lanka, etc. where self-immolation is common (13, 14). Self-burning as a fatal and gruesome mean of committing suicide (15) is believed to occur mainly through copycatting phenomenon (16-18).

Therefore, it would be necessary to determine that medical practitioners and medical students in the above-mentioned countries apply what type of methods to commit suicide. This should help to reveal that what proportion of suicides in these two groups could be attributable to familiarity with and easy access to dangerous means of suicide, that is, drugs and what proportion to copycat phenomenon, that is, self-immolation.

## Footenote

**Declaration of interest:** None.
